# Epigenetic regulation of cyclooxygenase-2 by methylation of c8orf4 in pulmonary fibrosis

**DOI:** 10.1042/CS20150697

**Published:** 2016-03-08

**Authors:** Iona C. Evans, Josephine L. Barnes, Ian M. Garner, David R. Pearce, Toby M. Maher, Xu Shiwen, Elisabetta A. Renzoni, Athol U. Wells, Christopher P. Denton, Geoffrey J. Laurent, David J. Abraham, Robin J. McAnulty

**Affiliations:** * UCL Respiratory Centre for Inflammation and Tissue Repair, Rayne Building, University College London, London, WC1E 6JF, U.K.; † NIHR Biomedical Research Unit & Interstitial Lung Disease Unit, Royal Brompton Hospital, Sydney Street, London, SW3 6NP, U.K.; ‡ Centre for Rheumatology and Connective Tissue Diseases, Royal Free Campus, University College London, London, NW3 2PF, U.K.; § Centre for Cell Therapy and Regenerative Medicine, School of Medicine and Pharmacology, University of Western Australia, Harry Perkins Institute of Medical Research, Crawley, WA 6009, Australia

**Keywords:** cyclooxygenase-2, DNA methylation, fibroblast, idiopathic pulmonary fibrosis, prostaglandin E_2_, systemic sclerosis

## Abstract

Fibroblasts derived from the lungs of patients with idiopathic pulmonary fibrosis (IPF) and systemic sclerosis (SSc) produce low levels of prostaglandin (PG) E_2_, due to a limited capacity to up-regulate cyclooxygenase-2 (COX-2). This deficiency contributes functionally to the fibroproliferative state, however the mechanisms responsible are incompletely understood. In the present study, we examined whether the reduced level of COX-2 mRNA expression observed in fibrotic lung fibroblasts is regulated epigenetically. The DNA methylation inhibitor, 5-aza-2′-deoxycytidine (5AZA) restored COX-2 mRNA expression by fibrotic lung fibroblasts dose dependently. Functionally, this resulted in normalization of fibroblast phenotype in terms of PGE_2_ production, collagen mRNA expression and sensitivity to apoptosis. COX-2 methylation assessed by bisulfite sequencing and methylation microarrays was not different in fibrotic fibroblasts compared with controls. However, further analysis of the methylation array data identified a transcriptional regulator, chromosome 8 open reading frame 4 (thyroid cancer protein 1, TC-1) (c8orf4), which is hypermethylated and down-regulated in fibrotic fibroblasts compared with controls. siRNA knockdown of c8orf4 in control fibroblasts down-regulated COX-2 and PGE_2_ production generating a phenotype similar to that observed in fibrotic lung fibroblasts. Chromatin immunoprecipitation demonstrated that c8orf4 regulates COX-2 expression in lung fibroblasts through binding of the proximal promoter. We conclude that the decreased capacity of fibrotic lung fibroblasts to up-regulate COX-2 expression and COX-2-derived PGE_2_ synthesis is due to an indirect epigenetic mechanism involving hypermethylation of the transcriptional regulator, c8orf4.

## Abstract (precis)

The present study demonstrates that hypermethylation and silencing of chromosome 8 open reading frame 4 (thyroid cancer protein 1, TC-1) (c8orf4), a transcriptional regulator of cyclooxygenase-2 (COX-2), is a major contributor to failure of fibroblasts to up-regulate COX-2 in pulmonary fibrosis. DNA methyltransferase (DNMT) inhibition reduces c8orf4 methylation, restores COX-2 expression and normalizes fibroblast function.

## CLINICAL PERSPECTIVES

• Reduced fibroblast expression of COX-2 and consequently low levels of PGE_2_ are significant contributors to the fibroproliferation that characterizes pulmonary fibrosis. The mechanisms underlying this failure to up-regulate COX-2 are incompletely understood.
• The present study demonstrates that the diminished capacity of IPF and SSc lung fibroblasts to up-regulate COX-2 results from aberrant epigenetic regulation. Hypermethylation and silencing of c8orf4, a transcriptional regulator of COX-2 in fibrotic lung fibroblasts, is a major contributor to their failure to up-regulate COX-2. Consistent with this, knockdown of c8orf4 in control fibroblasts induces a profibrotic phenotype.
• Targeting DNA methylation may represent a novel therapeutic approach for the treatment of pulmonary fibrosis.

## INTRODUCTION

Pulmonary fibrosis is a progressive disease that has a poor prognosis and for which there is currently no adequate therapy. It can occur in isolation, as in idiopathic pulmonary fibrosis (IPF) or in association with multi-organ connective tissue diseases, such as systemic sclerosis (SSc) [1,2]. In the most common and aggressive form, IPF, mean survival time from diagnosis is only 2–3 years [3].

The pathogenesis of pulmonary fibrosis is incompletely understood, although it is considered to develop through aberrant injury repair mechanisms with the fibroblast/myofibroblast a key effector cell [4,5]. We have previously reported that fibroblasts derived from the lungs of patients with IPF and SSc exhibit reduced capacity to up-regulate cyclooxygenase-2 (*COX-2*, *PTGS2*) and its downstream anti-fibrotic product prostaglandin (PG) E_2_, compared with control lung fibroblasts, and that this imparts a functionally profibrotic phenotype to the cells [6]. In addition, the inability to up-regulate COX-2 and PGE_2_ has been demonstrated to contribute to reduced HGF production by IPF fibroblasts [7]. The fundamental importance of COX-2 and PGE_2_ as anti-fibrotic mediators is supported by animal studies, demonstrating that genetic *COX-2* deficiency or COX inhibition enhances fibrotic responses in the lung [8–10]. Similarly, enhanced fibrotic responses in Granulocyte-macrophage colony stimulating factor (GM-CSF) deficient mice are associated with diminished PGE_2_ production [10,11]. Conversely, protection from fibrosis in animals deficient in CCR2, 5-lipoxygenase or transgenic overexpression of *TNFα* is at least partly attributable to up-regulation of PGE_2_ [12–15]. Together, these data demonstrate the critical importance of COX-2 and PGE_2_ in preventing the development of pulmonary fibrosis in animal models and human disease. However, the mechanisms responsible for limited *COX-2* expression in fibroblasts in the lungs of patients with pulmonary fibrosis are incompletely understood.

Accumulating evidence suggests that altered epigenetic marks such as DNA hypermethylation and histone hypoacetylation contribute to the silencing of anti-fibrotic genes in IPF lung and SSc skin fibroblasts [16,17], the development of renal and radiation-induced skin fibrosis [18,19] and fibroblast to myofibroblast differentiation [20]. Furthermore, *COX-2* promoter methylation and histone deacetylation have been implicated in *COX-2* gene silencing in cancer [21]. Recent studies examining DNA methylation in whole human lung tissue and cultured lung fibroblasts have shown multiple differentially methylated genes in IPF [22–24]. However, there is little information on the role of altered DNA methylation in the regulation of *COX-2* in IPF and SSc lung fibroblasts.

We hypothesized that DNA hypermethylation may contribute directly or indirectly to silencing of COX-2 expression in fibrotic lung fibroblasts. We show that treatment with a DNA methyltransferase (DNMT) inhibitor increased *COX-2* expression of fibrotic lung fibroblasts towards control levels, restored responsiveness to COX-2/PGE_2_ inducing agents and normalized fibroblast function. Although *COX-2* was found not to be directly methylated, we identified a *COX-2* binding transcriptional regulator, chromosome 8 open reading frame 4 (thyroid cancer protein 1, TC-1) (c8orf4), that is hypermethylated and down-regulated in fibrotic lung fibroblasts; knockdown of which in control fibroblasts induced a cell phenotype similar to that associated with fibrotic lung fibroblasts.

## MATERIALS AND METHODS

### Cell culture

Fibrotic lung tissue was obtained from either transplant surgery or lung biopsies and control tissue from histologically normal areas of peripheral lung removed at lung cancer resection, as previously described [25]. Primary fibroblast cultures were established as previously described [6] and used before passage 8. Patient details: Control, *n*=7, aged 56±6 years, 2 male; IPF, *n*=10, aged 64±3 years, 5 male; SSc, *n*=8, aged 52±1 years, 1 male). All tissue was obtained with appropriate consent and its use approved by the relevant research ethics committee.

Cells were routinely plated in 6-well plates in Dulbecco's modified Eagle's medium (DMEM) supplemented with 10% (v/v) FCS, 100 unit/ml penicillin and 100 mg/ml streptomycin (Invitrogen) and cultured at 37°C in a humidified atmosphere of air containing 10% CO_2_. Once confluent, the medium was removed and replaced with 0.4% FCS containing medium without antibiotics, and incubated for 24 h. Medium containing transforming growth factor-β_1_ (TGF-β_1_) (1 ng/ml final concentration) was added and incubated for up to 24 h before harvesting. For mRNA processing studies, following 3 h incubation with TGF-β_1_, actinomycin D, a transcription inhibitor, was added at a final concentration of 3 μg/ml to the existing medium, and cells harvested 0–75 min later for RNA extraction.

For the epigenetic studies, cells were seeded in 6-well plates at less than 30% confluence 24 h before treatment. 5-Aza-2′-deoxycytidine (5AZA), a DNMT inhibitor (Sigma–Aldrich), was added at the indicated concentrations to the culture medium and replaced at 24 h intervals for 3 days. Cells were then placed in 0.4% FCS medium containing 5AZA at the same concentration with or without trichostatin A (TSA), an inhibitor of histone deacetylases, at a concentration of 300 nM (Sigma–Aldrich) for a further 24 h as described previously [21]. Cells were then harvested for total RNA or DNA extraction.

For microarray analysis and bisulfite sequencing experiments, 500,000 cells were seeded in a T175 flasks, 24 h before treatment with or without a daily dose of 1 μM 5AZA until cells were confluent (≥1 week, to ensure a minimum of 3 population doublings). DNA was extracted (Nucleon, SL8501) and bisulfite converted as described below.

### qPCR

RNA was isolated using TRI-Reagent (Sigma–Aldrich) and DNase treated with DNA-free (Ambion). cDNA was synthesized using qScript (Quanta Biosciences). qPCR was performed using MESA GREEN qPCR MasterMix Plus for SYBR® Assay (Eurogentec) on an Eppendorf Realplex^4^ Mastercycler. Cycling conditions were as follows: 95°C for 5 min; and 40 cycles of 95°C (10 s) and 62°C (45 s). Data were normalized to either 18S or a combination of *HPRT*, *YWHAZ* and *EIF4A2* (as determined by geNorm). Primers detailed in Supplementary Table S1.

### PGE_2_ quantification

PGE_2_ was measured using a Biotrak Enzyme-immunoassay (GE Healthcare) according to the manufacturer's instructions.

### Induction and detection of apoptosis

Fas ligand (FasL)-induced apoptosis was measured in fibroblasts treated with or without 5AZA, as previously described [25].

### DNA methylation analysis

DNA extracted from fibroblasts treated with or without 5AZA was bisulfite converted using EZ DNA Methylation-Gold™ (Zymo Research). Bisulfite converted DNA was analysed using Illumina Infinium Human Methylation 450 array, bisulfite sequencing or pyrosequencing.

### Bisulfite sequencing

The DNA samples were bisulfite converted using an EZ DNA Methylation-Gold™ Kit (Zymo Research). PCR was performed on a tetrad PTC-225, Peltier Thermal cycler. PCR cycling conditions were: 94°C for 5 min, followed by 10 cycles of 94°C for 20 s, touchdown from 60°C to 50°C (−1°/cycle) for 20 s and 72°C for 30 s, followed by a further 35 cycles at 50°C annealing temperature. PCR products were resolved on a 1% agarose gel and PCR products purified using a QIAquick gel extraction kit (Qiagen, Germany) prior to DNA sequencing (WIBR, UCL). The relative methylation at each CpG site was determined by area under the curve analysis using ImageJ software, as previously described [26]. Primer details are provided in Supplementary Table S1.

### Pyrosequencing

Cells were seeded in T75 culture flasks and treated as described above with 10 μM 5AZA. DNA was extracted and bisulfite converted, as described above. Pyrosequencing assays were designed using the PyroQ assay design software. A common tag was placed on either the forward or reverse primer (depending on the strand to be sequenced) and a common universal biotinylated primer was used for all reactions as previously described [27]. PCR was performed using a nested PCR for specific amplification and cycling conditions included denaturation at 95°C for 4 min, followed by 10 cycles of 94°C for 15 s, touchdown from 60°C to 50°C (−1°/cycle) for 15 s and 72°C for 20 s, followed by a further 30 cycles at 50°C annealing temperature. The second PCR used 2 μl of a 1:10 dilution of the first PCR as template and the same cycling conditions. All products were confirmed to be single bands by agarose gel electrophoresis. Methylation values were calculated as an average of all 10 CpG sites within each assay as determined by the Pyro Q-CpG Software (Biotage). Primers detailed in Supplementary Table S2.

### Immunohistochemistry

Immunohistochemical staining was performed on formalin-fixed, paraffin-embedded sections of human lung tissue using the avidin–biotin antibody complexing method, as previously described [28]. c8orf4 and rabbit IgG antibodies used were ab121923 and ab27478 (Abcam).

### siRNA knockdown

Cells were seeded and transfected using INTERFERin (Polyplus Transfection), following the manufacturer's instructions. c8orf4 siRNA or negative control siRNA (#4392420 and #4390846, Ambion) were used at 10 nM. RNA and protein were isolated for qPCR and Western blot analysis.

### Western blotting

Twenty micrograms of protein was resolved on a 12.5% polyacrylamide gel, transferred on to a PVDF membrane and immuno-blotted for COX-2 and β-actin. Antibodies used: ab52237, Abcam; A5441, Sigma–Aldrich; P0448 and P0260, Dako, Glostrup.

### ChIP

ChIP was performed as previously described [29]. Briefly, cells were fixed in 1% formaldehyde for 15 min at room temperature and then quenched with 0.125 M glycine for 5 min. To generate DNA fragments of 0.2–1 kb, 1×10^6^ cells were sonicated by Bioruptor™ (Diagenode) on maximum power for 8 min with an on–off interval of 30 s. Samples were pre-cleared with Protein A agarose/Salmon Sperm DNA 50% Slurry (16–157, Millipore), before being immunoprecipitated with 3.5 μg of c8orf4 antibody (ab133885, Abcam) overnight at 4°C. For negative/isotype control rabbit IgG was used (ab27478, Abcam). Agarose/antibody/histone complexes were collected and washed before elution of protein/DNA complexes. Cross-linking of products was reversed by heating overnight at 65°C and then treated with proteinase K at 45°C for 1 h. DNA was recovered by QIAquick purification kit (Qiagen). DNA was amplified by PCR using a panel of *COX-2* promoter primer sets (Supplementary Table S1). PCR cycling conditions were: 94°C for 5 min; followed by 40 cycles of 94°C (1 min), 58°C (1 min) and 72°C (1 min); and 72°C for 5 min. PCR products were visualized on a 1.5% agarose gel.

### Statistical analysis

Statistical analysis was performed using Graphpad Prism (GraphPad Software). Data were evaluated using either one-way ANOVA with Tukey's post hoc pair wise comparisons or two-way ANOVA with a Bonferroni post-test. For microarray data, an adjusted *P* value of <0.05 and a Δ*β* value ≥0.136 was considered significant [30].

## RESULTS

### IPF- and SSc-derived fibroblasts exhibit a limited capacity for COX-2 mRNA transcription

**Figure 1:**
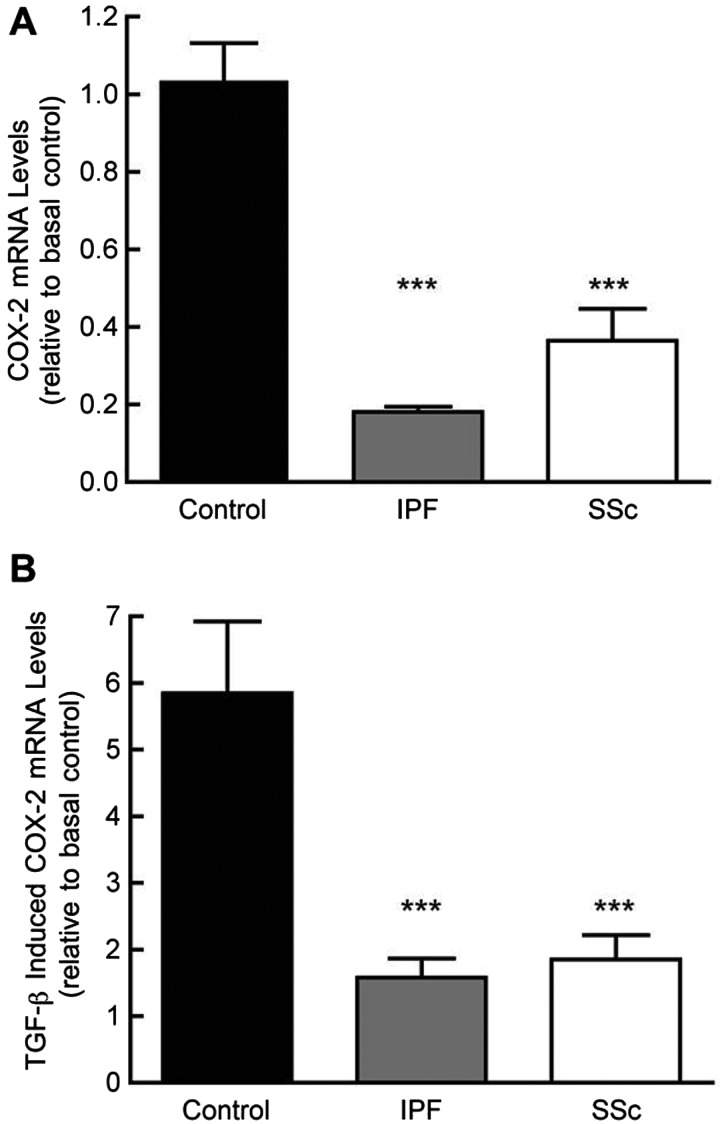
Basal and TGF-β-induced levels of COX-2 mRNA in control and fibrotic lung fibroblasts Fibroblasts were grown to confluence, serum deprived for 24 h, and then incubated for 3 h in medium alone (**A**) or in TGF-β_1_ at a concentration of 1 ng/ml (**B**), before RNA was extracted for quantitative real-time PCR. Data are enumerated as mRNA levels relative to mean basal levels in control cell lines. Each bar represents the mean ± S.E.M. for four control, seven IPF and four SSc donor cell isolates. (***P<0.001 compared with control, one-way ANOVA with Tukey's post-test).

Consistent with previous results [6,31], fibroblasts from IPF and SSc groups produced three- to five-fold lower basal levels of mature *COX-2* mRNA compared with controls (Figure 1A). Similar differences between groups were observed in TGF-β_1_-induced levels of *COX-2* mRNA (Figure 1B). *COX-2* mRNA levels in the control group increased approximately six-fold in response to 3 h stimulation with TGF-β_1_ (*P*<0.001). *COX-2* mRNA levels also increased in response to TGF-β_1_ in both the fibrotic groups; however, induced levels were still approximately three-fold lower than in controls (*P*<0.001). Additional studies comparing levels of the mature and nascent *COX-2* transcript revealed an almost identical time-dependent response to TGF-β_1_, with significantly lower levels of both nascent and processed transcripts seen in the fibrotic fibroblasts compared with control (Supplementary Figures S1A and S1B). Further, no differences were observed at the level of mRNA stability, when actinomycin D chase experiments were performed (Supplementary Figure S1C), thus suggesting that the differences in mRNA expression between control and fibrotic lung fibroblasts result from deficiencies in *COX-2* transcription.

### DNA demethylation but not histone acetylation restores fibrotic lung fibroblast *COX-2* expression

**Figure 2:**
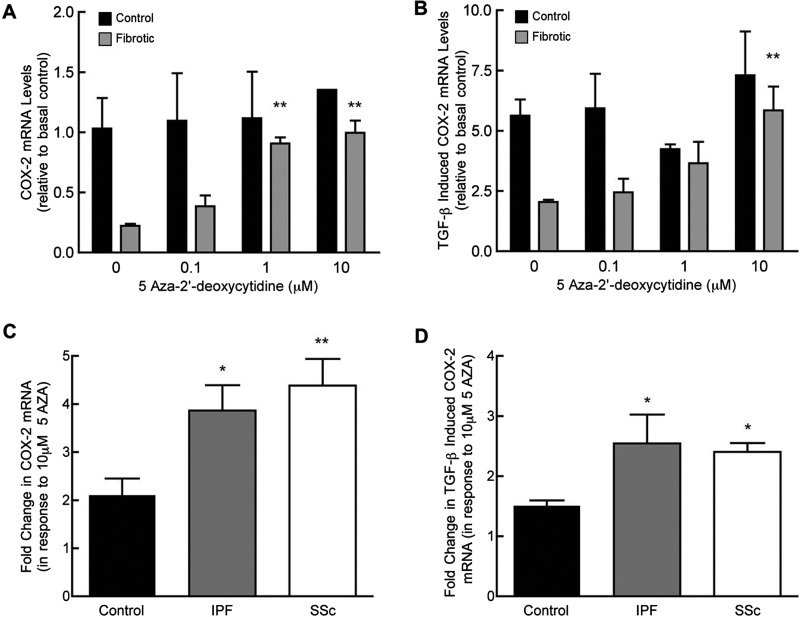
DNMT inhibition preferentially increases COX-2 mRNA levels in fibrotic lung fibroblasts Proliferating cultures of control and fibrotic lung fibroblast lines were incubated with indicated concentrations of 5AZA for 96 h. Fibroblasts were then treated with medium alone (**A**) or with TGF-β_1_ (1 ng/ml) for 3 h (**B**) before being harvested for COX-2 quantitative real-time PCR. Data are enumerated as the fold increase, relative to mean mRNA levels basally in control cells. Each bar represents the mean of duplicate samples ± S.E.M. for one representative control and fibrotic cell line (**P<0.01 compared with 0 μM 5AZA, two-way ANOVA with Bonferroni post-test). Proliferating cultures were incubated with 10 μM of 5AZA for 96 h and then treated with medium alone (**C**) or with TGF-β_1_ (1 ng/ml) for 3 h (**D**) before being harvested for COX-2 quantitative real-time PCR. Data are enumerated as the fold increase, relative to mean levels in non-5AZA treated cells. Each bar represents mean ± S.E.M. for four control, seven IPF and four SSc donor cell isolates (*P<0.05, **P<0.01, compared with control group, one-way ANOVA with Tukey's post-test).

To examine whether *COX-2* transcription is regulated epigenetically, cells were treated with the DNMT inhibitor, 5AZA or HDAC inhibitor, TSA. Treatment of representative control and fibrotic cell lines with 5AZA (0–10 μM), demonstrated a distinct difference in response. Demethylation did not affect *COX-2* expression in control fibroblasts (Figure 2A). In contrast, treatment with 5AZA resulted in a dose-dependent increase in *COX-2* expression in fibrotic lung fibroblasts, with levels higher at both 1 and 10 μM 5AZA compared with untreated cells (*P*<0.01, in both cases), and were similar to control. These data were confirmed by treatment of selected IPF and SSc fibroblasts with an alternative DNMT inhibitor, Zebularine, which also increased *COX-2* mRNA approximately six-fold (Supplementary Figure S2). Cells treated with 5AZA and TGF-β_1_ showed a similar pattern of response (Figure 2B). Relative levels of TGF-β_1_-induced *COX-2* mRNA were three-fold lower in fibrotic lung fibroblasts compared with the control cell line, in the absence of 5AZA. Fibrotic fibroblast COX-2 mRNA levels increased in a dose-dependent manner in response to 5AZA (*P*<0.05) with expression increased greater than two-fold at 10 μM (*P*<0.01). At the 10 μM dose of 5AZA, levels of COX-2 mRNA in the fibrotic cell line were similar to that seen in control fibroblasts. Similar dose-dependent responses were seen in other IPF- and SSc-derived cell lines (results not shown). Group data for control, IPF- and SSc-derived lung fibroblasts treated with 10 μM 5AZA is shown in Figure 2C. DNA demethylation increased COX-2 expression in control cell lines by two-fold (*P*<0.05). In contrast, both IPF and SSc fibroblast lines showed significantly greater increases (four-fold) in COX-2 expression in response to 5AZA compared with controls. Similar results were observed following stimulation with TGF-β_1_ although the differences between control and fibrotic groups were less marked (Figure 2D).

Previous studies have suggested that histone hypoacetylation contributes to the dysregulation of COX-2 expression in IPF lung fibroblasts [32]. Using TSA, we observed a trend towards increased COX-2 mRNA expression compared with untreated cells, in control and fibrotic fibroblasts, but this was not significant (Supplementary Figure S3A). Similar results were observed following TGF-β_1_ treatment, with no significant difference observed between the fibrotic and control groups (Supplementary Figure S3B).

### DNA demethylation restores fibrotic lung fibroblast function

**Figure 3:**
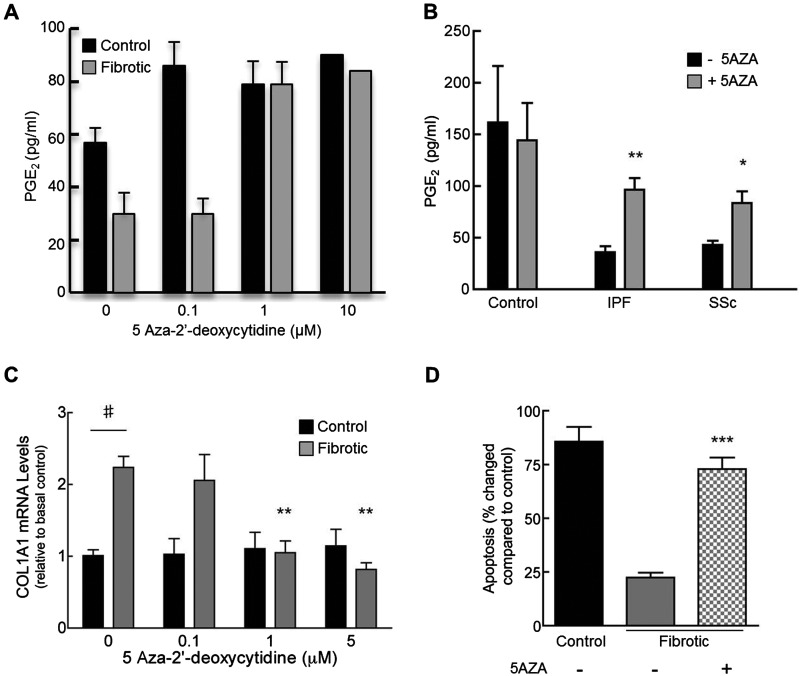
DNMT inhibition restores fibrotic lung fibroblast PGE_2_, collagen production and sensitivity to FasL-induced apoptosis Proliferating cultures of single representative control and fibrotic lung fibroblast isolates were treated with 0, 0.1, 1 or 10 μM 5AZA for 96 h. Cells were serum deprived for 24 h before cell supernatants were harvested for PGE_2_ enzyme immunoassay. (**A**) Each bar represents the mean of duplicate samples ± S.E.M. (where no error bar is shown, only one sample was available for analysis). Cells from each group (control; *n*=4, IPF; *n*=7, SSc; *n*=4) were grown with 10 μM 5AZA before cell supernatants were collected for measuring PGE_2_ production (**B**). Each bar represents the mean ± S.E.M. (*P<0.05, **P<0.01, compared with control group, two-way ANOVA with Bonferroni post-test). Representative control and fibrotic lung fibroblasts were incubated with 5AZA before being harvested for collagen 1A qPCR (**C**). Data are enumerated as the fold increase, relative to mean mRNA levels basally in control cells. Each bar represents the mean ± S.E.M. for three independent experiments (**P<0.01 compared with 0 μM 5AZA, two-way ANOVA with Bonferroni post-test. ^#^*P*<0.01 compared with basal levels in control). Representative control and fibrotic lung fibroblasts were grown in the presence of either 10 μM 5AZA or vehicle alone, in medium containing 10% FBS for 72 h (**D**). Fibroblasts were then serum starved overnight with or without 5AZA prior to being exposed to FasL (50 ng/ml) for 24 h. Apoptosis was determined by Annexin V/PI staining with FACS analysis. Bars represent the mean ± S.E.M. for six experimental replicates (***P<0.001 compared with non-5AZA treated, two-way ANOVA with Bonferroni post-test).

To investigate whether the up-regulation of *COX-2* mRNA seen in fibrotic lung fibroblasts following treatment of cells with 5AZA had functional downstream consequences, we measured the level of PGE_2_, in cell supernatants (Figure 3A). Similar to the effects of 5AZA on *COX-2* mRNA, two-way ANOVA demonstrated a statistically significant difference in PGE_2_ production between control and fibrotic lung fibroblasts (*P*<0.05) and a dose-dependent response to demethylation was also observed in fibrotic fibroblast PGE_2_ production (*P*<0.005). Group data shows that basal levels of PGE_2_ produced by control cells are significantly higher than levels produced by both the fibrotic groups (*P*<0.01, compared with IPF and *P*<0.05, compared with SSc) (Figure 3B). Treatment of cells with 5AZA resulted in approximately three- and two-fold increases in PGE_2_ production in IPF- and SSc-derived fibroblasts respectively (*P*<0.001 and *P*<0.02 respectively). PGE_2_ production by control cell lines was not altered by treatment with 5AZA. Mean levels of PGE_2_ produced by both the fibrotic groups following treatment with 5AZA were not significantly different from those produced by controls.

Quantitative real-time PCR was used to analyse levels of collagen mRNA production in both control and fibrotic lung fibroblasts. Basal levels of collagen 1A mRNA in IPF fibroblasts were two-fold higher compared with control (Figure 3C). Treatment with 5AZA did not affect collagen 1A expression in control cells; whereas a dose-dependent and significant decrease in collagen 1A mRNA levels was observed in IPF fibroblasts (*P*<0.01). At the higher doses of 5AZA, there was no significant difference between the collagen 1A expression measured in control and fibrotic cells.

In agreement with our previous data [25], we demonstrate that fibrotic lung fibroblasts are resistant to FasL-induced apoptosis when compared with control (Figure 3D). Growth of fibrotic cells with 5AZA led to a greater than three-fold increase in the sensitivity of these cells to FasL-induced apoptosis, compared with non-AZA treated cells (*P*<0.001), resulting in levels comparable to that seen in control cells.

### The COX-2 promoter is not differentially methylated in fibrotic lung fibroblasts

In order to examine whether the effects observed in fibrotic lung fibroblasts in response to 5AZA treatment, are a consequence of a direct effect on *COX-2*, we used pyrosequencing to investigate the methylation status of CpG sites within the *COX-2* promoter. Twenty-seven CpG sites were analysed in three different regions (Supplementary Figure S4A), two of which were previously identified as being methylation sensitive and having a functional role in regulating COX-2 expression [33,34]. However, our data demonstrate that none of the CpG sites/regions were hypermethylated in control or fibrotic lung fibroblasts (Supplementary Figure S4B). Levels of methylation in both 5AZA and TGF-β_1_ treated cells were also analysed and demonstrated no significant differences (results not shown). These results were further confirmed by methylation microarray analysis (Supplementary Figure S4C). Seventeen CpG sites were analysed (five of which were previously assessed by pyrosequencing) and no differential methylation was observed at any position.

### Identification of c8orf4 as a novel COX-2 regulator in pulmonary fibrosis

**Figure 4:**
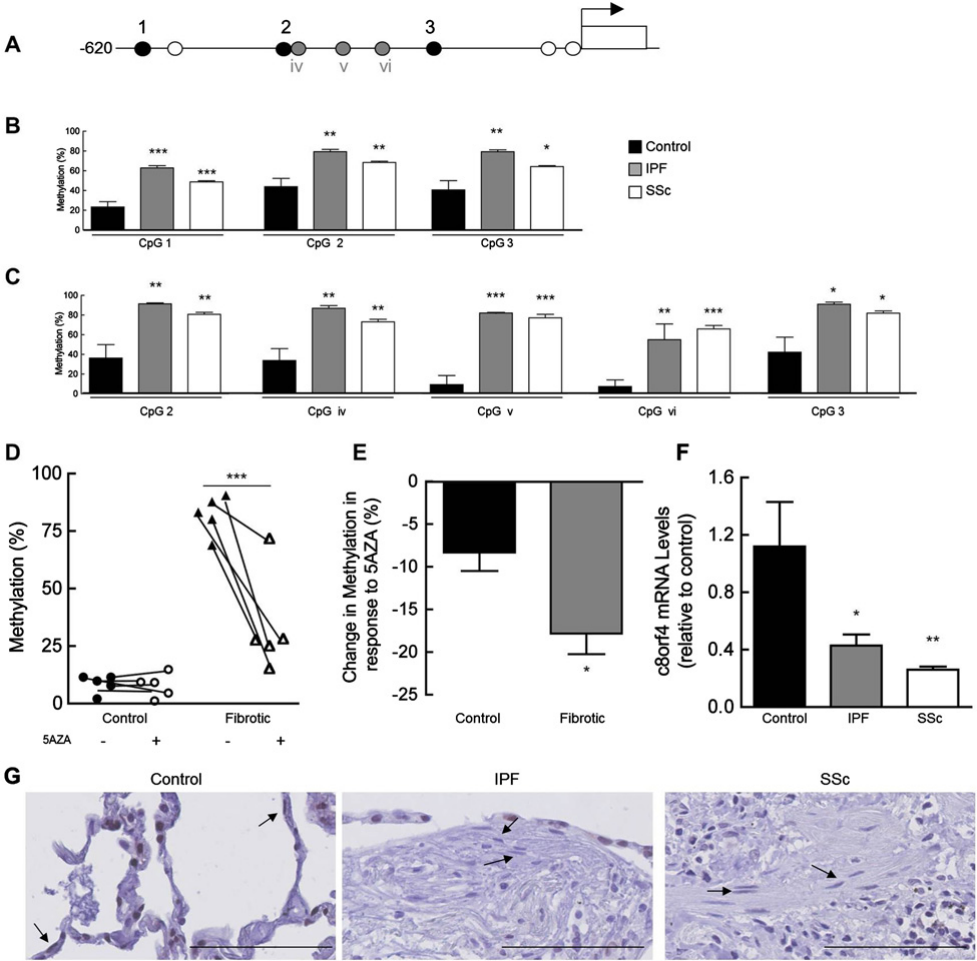
Increased methylation and decreased expression of c8orf4 in fibrotic lung fibroblasts A schematic representation of the upstream 5′ region of c8orf4 (**A**). Circles indicate the location of CpG sites. Black circles represent the three CpG sites on the Illumina array, grey circles represent three additional sites tested by bisulfite sequencing and white circles represent untested CpG sites within the region. Genomic DNA extracted from primary fibroblasts was assessed and mean percentage methylation at each CpG site within each group is shown (**B**). Genomic DNA extracted from control, IPF and SSc cell lines were bisulfite treated and subjected to bisufite sequencing (**C**). Five CpG sites within the c8orf4 promoter were analysed. Mean percentage methylation at each CpG site within each of the groups is shown (**C**). For (B, C, E and F) data are derived from six control, five IPF and seven SSc donor fibroblast isolates. ***P<0.001, **P<0.01 and *P<0.05, compared with levels in control cells, one-way ANOVA with Tukey's post-test. DNA extracted from representative control and fibrotic fibroblasts treated with or without 1 μM 5AZA for ≥1 week was subjected to bisufite sequencing, as above (**D**). Each point represents the % methylation at the five individual CpG sites analysed in panel C (***P<0.001 compared with non-5Aza treated, two-way ANOVA with Bonferroni post-test). Control, IPF and SSc lung fibroblast lines were treated with 1 μM 5AZA as above, and DNA subjected to bisulfite sequencing (**E**). Data are enumerated as mean % DNA methylation across all five CpG sites within each group (*P<0.05, compared with levels in control cells). RNA extracted from control and fibrotic cell lines was subjected to qPCR for c8orf4 mRNA (**F**). Data are enumerated as mRNA levels relative to mean basal levels in control cells (±S.E.M., **P<0.01 and *P<0.05, one-way ANOVA with Tukey's post-test). Representative photomicrographs of control (*n*=3), IPF (*n*=6) and SSc (*n*=1) lung showing immunohistochemical localization of c8orf4 stained brown/black (**G**). Arrows indicate nuclear c8orf4 staining in control lung fibroblast-like cells but staining was weak or undetectable in fibrotic lung fibroblasts (scale bar represents 50 μm).

The Illumina Infinium Human Methylation 450 array was used to identify genes with altered methylation in IPF and SSc fibrotic lung fibroblasts compared with control, and to identify genes that may epigenetically regulate *COX-2*. The array contains three probes corresponding to the promoter region of *c8orf4* (Figure 4A). All three CpG sites were hypermethylated in all IPF (*n*=5) and SSc (*n*=7) fibroblasts compared with control (*n*=6); with on average a 24–40% higher methylation level in fibrotics compared with control, across all three sites (Figure 4B). Methylation at two of these CpG sites (CpG 2 and 3) was confirmed by bisulfite sequencing (Figure 4C). A further three CpG sites (CpG iv, v and vi) situated between CpG 2 and CpG3 were also analysed by bisulfite sequencing (grey circles, Figure 4A). Methylation levels at all five sites sequenced, were on average 40–70% higher in both SSc and IPF compared with control (Figure 4C).

Treatment of fibrotic lung fibroblasts with 1 μM 5AZA resulted in a significant decrease in methylation levels at all five CpG sites analysed within the *c8orf4* promoter (Figure 4D). Bisulfite sequencing data from representative control and fibrotic cell lines shows no significant change in methylation in control fibroblasts after treatment with 5AZA, at any of the CpG sites. However, in fibrotic fibroblasts, methylation decreased by up to 65%. Group data shows a significantly greater decrease in mean DNA methylation over all CpG sites in the fibrotics compared with controls (Figure 4E). The mean DNA methylation in the controls was not significantly different from that in non-5AZA treated cells. However, on average, an 18% decrease in mean DNA methylation was observed, compared with non-5AZA treated fibrotic lung fibroblasts.

Quantitative real-time PCR was used to assess whether differences in methylation levels had any effect on the expression of *c8orf4*. Our results demonstrated that both IPF and SSc fibroblasts on average had 2.5- and 5.5-fold lower levels, respectively, of *c8orf4* mRNA compared with control fibroblasts (Figure 4F). In keeping with this finding, immunohistochemical staining for c8orf4 on paraffin-embedded sections of human lung tissue, shows intense nuclear staining in the control fibroblasts (Figure 4G), whereas staining for c8orf4 in fibroblasts of fibrotic lung was undetectable.

**Figure 5:**
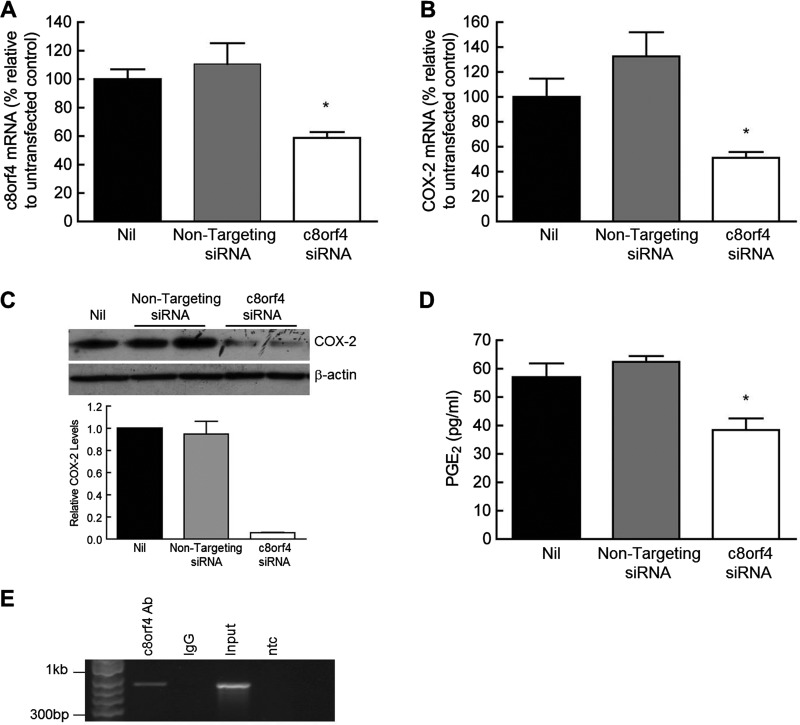
c8orf4 associates with the COX-2 promoter and knockdown in control fibroblasts down-regulates expression of COX-2 and PGE_2_ production Control fibroblasts were transfected with either a non-targeting negative control or c8orf4 siRNA (10 nM) and RNA was extracted for qPCR (**A** and **B**). Levels of c8orf4 (**A**) and COX-2 (**B**) mRNA are represented as a percentage relative to non-transfected cells (Nil). Each bar represents mean ± S.E.M. for three independent experiments. (*P<0.05 compared with Nil, one-way ANOVA with Tukey's post-test). Protein samples were collected from cells transfected as above, and samples Western blotted for COX-2 (**C**). Relative COX-2 levels are expressed as a ratio of COX-2/β-actin and represented relative to levels in non-transfected cells. Cells were transfected as above, and cell supernatants were harvested for PGE_2_ enzyme immunoassay (**D**). Each bar represents the mean of triplicate wells ± S.E.M. and is representative of three individual experiments (*P<0.05 compared with non-transfected control, one-way ANOVA with Tukey's post-test). Chromatin extracted from representative primary control fibroblasts was immunoprecipitated with a c8orf4 antibody followed by PCR with a panel of COX-2 promoter primer sets (**E**). ChIP PCR products, corresponding to region −579 to −1271, were visualized by agarose gel electrophoresis (IgG corresponds to isotype control; input is sonicated but non-immunoprecipitated sample and ntc is no template control for the PCR).

To examine whether c8orf4 regulates *COX-2* transcription, control fibroblasts were transfected with *c8orf4* siRNA. A 40% knockdown of *c8orf4* mRNA was achieved with the *c8orf4* siRNA, compared with non-transfected cells (Figure 5A). Transfection with a non-targeting negative control siRNA, had no significant effect on levels of *c8orf4* mRNA (ANOVA). Knockdown of *c8orf4* additionally resulted in a 50% knockdown of *COX-2* mRNA, compared with non-treated cells with no significant effects on *COX-2* mRNA levels using a non-targeting control siRNA (Figure 5B). Furthermore, Western blotting siRNA transfected cell lysates using a COX-2 antibody, demonstrated an almost complete (95%) knockdown of the COX-2 protein in cells transfected with *c8orf4* siRNA (Figure 5C). Analogous to the effect seen on COX-2, transfection of cells with *c8orf4* siRNA further resulted in a 35% decrease in PGE_2_ production, compared with non-treated cells (Figure 5D), whereas transfection with non-targeting control siRNA had no effect. In order to determine whether c8orf4 is able to transcriptionally regulate COX-2, we used chromatin immunoprecipitation to assess whether it was able to bind to the *COX-2* promoter. A panel of COX-2 promoter primer sets spanning region +54 to −3654 was used to amplify *COX-2* DNA bound by the c8orf4 antibody (results not shown). ChIP revealed the binding of endogenous c8orf4 to the *COX-2* promoter, at region −579 to −1271 (Figure 5E).

## DISCUSSION

We and others have previously demonstrated that pulmonary fibroblasts derived from the lungs of patients with IPF and SSc express significantly lower steady-state levels of *COX-2* RNA, compared with controls, basally and in response to TGF-β_1_ [6,31,32]. In the present study, we confirm this using quantitative real-time PCR for mature COX-2 mRNA transcripts and additionally demonstrate, by measuring the production of nascent COX-2 transcripts and use of actinomycin D, that the deficiency in *COX-2* expression in fibrotic lung fibroblasts is at the level of transcription and is not due to altered RNA processing or stability.

Epigenetic regulation and in particular DNA methylation is known to play a critical role in the transcriptional silencing of genes [35]. Its role in tumorigenesis is well recognized, and there is increasing recognition of a role in other disease settings, including pulmonary fibrosis. It has been demonstrated that hypermethylation corresponds to the reduced expression of numerous genes in IPF and SSc fibroblasts [16,17,36–38]. These include the *PTGER2* gene, which encodes the E prostanoid 2 receptor (EP2), which is known to be a major receptor responsible for anti-fibrotic effects of PGE_2_ [37,39]. We demonstrate that the treatment of IPF and SSc fibroblasts, but not control fibroblasts, with 5AZA resulted in a concentration-dependent increase in *COX-2* expression, PGE_2_ production, decreased collagen expression, as well as restoring sensitivity to apoptosis in fibrotic lung fibroblasts. Together, these data indicate that DNA hypermethylation of COX-2/PGE_2_ pathway components may play a crucial role in the dysregulated synthesis of this critical anti-fibrotic mediator, and contribute to pathogenesis of pulmonary fibrosis.

Hypermethylation of the *COX-2* promoter has previously been shown to be responsible for gene silencing in cancer [21,33,34]. However, we found no evidence of CpG hypermethylation in the *COX-2* promoter or other gene regions tested, suggesting a different mechanism for *COX-2* silencing in lung fibrosis to that observed in cancer [33,34]. Consequently, this suggested an indirect epigenetic mechanism for down-regulation of *COX-2* expression in fibrotic lung fibroblasts. One potential explanation could be that a transcription factor or co-stimulatory molecule is hypermethylated, preventing *COX-2* transcription. Through microarray based screening of gene methylation, we discovered a COX-2 regulatory factor, c8orf4, which is hypermethylated and has reduced expression in both IPF and SSc lung fibroblasts. This is consistent with previous data showing hypermethylation of *c8orf4* resulting in its reduced expression in cancer [40]. In addition, we demonstrate binding of c8orf4 to the proximal *COX-2* promoter and that siRNA knockdown of c8orf4 in control fibroblasts reduced *COX-2* expression and PGE_2_ synthesis analogous with the phenotype of fibrotic lung fibroblasts.

*C8orf4* encodes a small, highly conserved, nuclear localized protein. Its structural characterization indicates that it performs pivotal functions in the area of cell cycle control as well as transcriptional and translational regulation [41,42]. c8orf4 has previously been linked to COX-2, where over-expression of *c8orf4* in human aortic endothelial cells resulted in up-regulation of COX-2 [43]. C8orf4, mainly studied in the context of cancer, has more recently been linked to local inflammation and immune responses [43–45]. In non-fibroblast cell types, it has been demonstrated that c8orf4 can be up-regulated by TGFβ, IL-1β, TNFα and LPS. These cytokines are known profibrotic mediators that are also known to up-regulate PGE_2_/COX-2 in control lung fibroblasts. Further, fibroblasts cultured from patients with IPF fail to induce PGE_2_ synthesis on stimulation with these mediators due to aberrant *COX-2* expression [6,31,32,46]. Together these data strongly suggest that hypermethylation and silencing of *c8orf4* inhibits the expression of *COX-2* and PGE_2_, basally and in response to profibrotic mediators, making an important contribution to the pathogenesis of pulmonary fibrosis.

It is becoming apparent that multiple epigenetic mechanisms are involved in the pathogenesis of fibrotic lung diseases. In addition to DNA methylation, defects in epigenetic mechanisms including histone modification, DNMT expression and miRNA expression have been implicated in the pathogenesis of pulmonary fibrosis. Contrary to previously published data [32], we observed a trend towards increased levels of COX-2 expression following HDAC inhibition in control and fibrotic lung fibroblasts but no clear differential between control or fibrotic lung fibroblasts, basally or in combination with TGF-β_1_. Coward et al. [32] report that combined HDAC inhibition and cytokine stimulation, resulted in increased *COX-2* expression in IPF lung fibroblasts. However, only 2 out of the 11 fibrotic cell lines we tested demonstrated an increased level of *COX-2* expression in response to HDAC inhibition and TGF-β_1_ stimulation. It would therefore appear that acetylation of histones may play a role in the regulation of COX-2 but it is unclear whether there are differences between control and fibrotic lung fibroblasts. Coward et al. also demonstrated that total HDAC activity was reduced in IPF fibroblasts, indicating that altered histone acetylation may play a wider role in increasing the activity of other genes involved in IPF. More recently, the same group have demonstrated that alterations in histone methylation also play a role in regulating *COX-*2 expression [47]. A recent publication by Dakhlallah et al. [48] has added another level of epigenetic regulation in IPF. They demonstrate that DNMT1 expression is regulated by several miRNAs. The emerging data from this and other studies suggests that altered epigenetic regulation is a complex but widespread and important phenomenon in pulmonary fibrosis.

In summary, we have identified a COX-2 regulatory factor, c8orf4, which is hypermethylated and down-regulated in fibrotic lung fibroblasts and tissue compared with controls. These findings add to the growing body of evidence which suggests that aberrant DNA methylation is an important pathogenic feature of pulmonary fibrosis. The reversibility of the epigenetic suppression on *COX-2* expression following DNMT inhibition, and normalization of fibroblast function, implies that it may be possible to reprogramme the phenotype of fibrotic fibroblasts, and as a result potentially improve the clinical outcome of patients with pulmonary fibrosis. DNA demethylation agents are already licensed for clinical use. Our studies, together with others strongly support the evaluation of epigenetically targeted therapies, possibly in combination with COX-2/PGE_2_ therapies for progressive fibrotic lung disease. The evaluation of such novel therapeutic approaches is necessary for what is currently a progressive disease with limited treatment options and a dismal prognosis.

## Abbreviations

5AZA: 5-aza-2′-deoxycytidine
c8orf4: chromosome 8 open reading frame 4 (thyroid cancer protein 1, TC-1)
COX-2: cyclooxygenase-2 (*PTGS2*, prostaglandin-endoperoxide synthase 2)
DNMT: DNA methyltransferase
FasL: Fas ligand
IPF: idiopathic pulmonary fibrosis
PG: prostaglandin
PGE_2_: prostaglandin E_2_
SSc: systemic sclerosis
TGF-β: transforming growth factor-β
TSA: trichostatin A
